# “Omics”-Informed Drug and Biomarker Discovery: Opportunities, Challenges and Future Perspectives

**DOI:** 10.3390/proteomes4030028

**Published:** 2016-09-12

**Authors:** Holly Matthews, James Hanison, Niroshini Nirmalan

**Affiliations:** 1 Department of Life Sciences, Faculty of Natural Sciences, Imperial College, London SW7 2AZ, UK; holly.matthews@imperial.ac.uk; 2 Manchester Royal Infirmary, Oxford Road, Greater Manchester M13 9WL, UK; James.Hanison@cmft.nhs.uk; 3 Environment and Life Sciences, University of Salford, Greater Manchester M5 4WT, UK

**Keywords:** drug discovery, omics, genomics, proteomics, metabolomics

## Abstract

The pharmaceutical industry faces unsustainable program failure despite significant increases in investment. Dwindling discovery pipelines, rapidly expanding R&D budgets and increasing regulatory control, predict significant gaps in the future drug markets. The cumulative duration of discovery from concept to commercialisation is unacceptably lengthy, and adds to the deepening crisis. Existing animal models predicting clinical translations are simplistic, highly reductionist and, therefore, not fit for purpose. The catastrophic consequences of ever-increasing attrition rates are most likely to be felt in the developing world, where resistance acquisition by killer diseases like malaria, tuberculosis and HIV have paced far ahead of new drug discovery. The coming of age of Omics-based applications makes available a formidable technological resource to further expand our knowledge of the complexities of human disease. The standardisation, analysis and comprehensive collation of the “data-heavy” outputs of these sciences are indeed challenging. A renewed focus on increasing reproducibility by understanding inherent biological, methodological, technical and analytical variables is crucial if reliable and useful inferences with potential for translation are to be achieved. The individual Omics sciences—genomics, transcriptomics, proteomics and metabolomics—have the singular advantage of being complimentary for cross validation, and together could potentially enable a much-needed systems biology perspective of the perturbations underlying disease processes. If current adverse trends are to be reversed, it is imperative that a shift in the R&D focus from speed to quality is achieved. In this review, we discuss the potential implications of recent Omics-based advances for the drug development process.

## 1. Introduction

Significant investments in drug discovery have failed to be accompanied by a parallel increase in the number of new molecular/biopharmaceutical entities (NMEs, NBEs) gaining regulatory approval by the US Food and Drug Administration (FDA). The burgeoning cost of drug development in a backdrop of diminishing productivity is likely to adversely impact the sustainability of the pharmaceutical industry [1]. In the past six decades, while the number of NMEs/NBEs launched every year has progressively declined, each decade has witnessed the progressive doubling of the inflation-adjusted cost of launching a drug [1,2,3]. Advancing a single drug from concept to market, when adjusted for post-approval Phase IV expenses, is estimated to exceed 1 billion US dollars [2]. The traditional drug discovery process itself continues to be tedious, costly, time consuming and very risky. The outcome of the unprecedented challenges facing the pharmaceutical industry is reflected by a generalised scarcity of late-stage R&D pipelines. Reducing the attrition rates and reversing the current productivity trends will require a multi-pronged initiative, namely, stringent and reliable target selection and validation, improved animal model systems and the definition of reliable biomarker and surrogate endpoints permitting early point of care studies in clinical trials.

The maturation of Omics-based technologies has meant that resulting data outputs, if systematically integrated, could have a significant impact on accelerating the drug discovery and development process by addressing some of the challenges mentioned above. Applications of Omics outputs have already been used in key areas of basic science underlying drug and biomarker discovery, although not to the extent originally speculated. Its contribution to enhancing our mechanistic understanding of the pathophysiology of disease processes cannot be disputed. However, early predictions grossly underestimated the maturation time for these complex, new sciences. The challenges inherent in quantifying and analysing enormous data outputs in a background of wide-ranging pre-analytical, methodological and technical variables resulted in erroneous interpretations which tainted the reputation of Omics-based research in general [4]. The expected increase in the chemical and biological spaces that could potentially be interrogated for therapeutic conjunctions failed to materialise [5] and reproducibility remains a critical issue hampering the successful translation of discovery research [6,7]. Arguably, the reasons for these failures are not confined to technical limitations. Biological pathways are complex, convoluted and interconnected. The daunting task of dissecting out and interpreting expression changes in a complex genome or proteome is bound to generate many false-positive and false-negative signals, even within a highly homogeneous, selected, sample cohort. Recent advances in chromatography, mass spectrometry, bio-computing and bioinformatics are significantly changing the Omics landscape and higher fidelity expression data is set to prove an indispensable tool for delivering mechanistic and predictive insights informing the process of drug discovery. Evidently, the vast biological variation within sample cohorts will continue to be a challenge. But in this arena, too, Omics-based technology is poised play a key role in the science underpinning drug discovery. By enabling biomarkers that permit the stratification of both patients and complex diseases, the method facilitates the identification of homogeneous patient subsets who are likely to deliver a higher chance of success in the clinical trial phase. Furthermore, outputs from the different Omics streams (genomics, transcriptomics, proteomics and metabolomics) have the singular advantage of complementarity, enabling cross-corroboration and validation.

The past decade has evidenced unprecedented challenges for the pharmaceutical industry resulting in unsustainable R&D costs. Predictably, Third-World diseases with poor returns on pharmaceutical investment are expected to be adversely impacted in the first instance. The global implications of a gap in the therapeutic market for major diseases like malaria and tuberculosis, which have failed to keep abreast of resistance acquisition, are dire [8].

In the early phase of drug discovery, much of the decisions around target selection, validation and lead generation are based on published literature, and hence the validity and reproducibility of such data is crucial to the successful progression of leads through the development pipeline. Despite substantial advances in early translational research, much of the knowledge derived to date is fragmented and piece meal, precluding a complete understanding of the complex biological interactions inherent in human disease. In the Omics arena, as with any new area of research, the transition to maturity has necessarily entailed a significant content of spurious data, which now has to be sieved through cautiously to extract the “paddy from the husk.” 

Nevertheless, the potential contribution of stringent, reproducible Omics-based data to drug discovery and development cannot be over-emphasised or indeed undervalued. While new guidelines for publishing Omics research (e.g., Paris guidelines for Proteomic data (http://www.mcponline.org/misc/ParisReport_Final.dtl [9]), have addressed some of these concerns, more work clearly needs to be done toward defining analytical variables that contribute to the lack of reproducibility among studies [4]. The shift in Omics-based research, particularly newer disciplines like metabolomics, from industrial to academic settings is indeed a redeeming and promising trend. The full potential of these powerful tools can only be realised in a setting where detailed and time-consuming optimisations are enabled, rather than when profit and returns are the driving impetus. An increasing realisation of this fact has renewed interest to forge, drive forward and exploit the benefits of unifying industrial and academic partnerships. If future investment is dedicated towards enabling key stakeholder participation (industry-academia-regulator consortia), there is little doubt that integration and implementation of newer Omics applications could provide novel, clinically valid targets and eventually the much-needed breakthrough medicines. Drug development is a complex and progressive discipline reliant on a diverse range of methodologies. Genomics, proteomics and metabolomics, encompassing new, rapidly advancing tools and techniques will continue to be increasingly employed in the development process. In this review, we focus on the contribution of emergent Omics-based biotechnologies, towards progressing the field of drug discovery and development.

## 2. Drug Development Pathways

**Figure 1:**
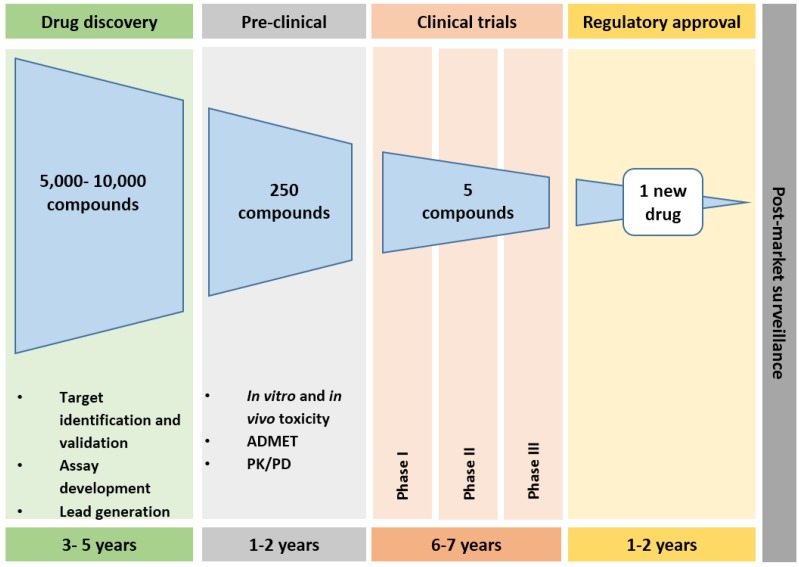
Drug discovery and development timeline. The current drug approval pipeline can take ~15 years. It is estimated that from 5,000–10,000 compounds only one new drug reaches the market. (Adapted from http://cmidd.northwestern.edu/files/2015/10/Drug_RD_Brochure-12e7vs6.pdf [10]; http://www.phrma.org/sites/default/files/pdf/rd_brochure_022307.pdf [11])

The drug development pathway for a small molecule entails an exhaustive process which includes basic research, target identification and validation, lead generation and optimisation, pre-clinical testing, phased-clinical trials in humans and regulatory approval by the FDA (Figure 1). The process of drug development begins with the identification of a novel druggable target (protein, DNA, RNA, metabolite, etc.) followed by its subsequent validation to confirm a therapeutic effect. This includes assay development/optimisation (biochemical, cell-based, cytotoxicity, etc.), whereby an objective methodology is derived to capture the intended interaction between a library of compounds and the specific target. Confirmed “hits” from high-throughput screens are then organised by chemical type to identify “leads” or chemical scaffolds which could be further refined by medicinal chemistry informed by structure-activity relationships (SAR), to optimise physicochemical and pharmacological properties for enhanced potency and selectivity.

Preliminary lead generation and optimisation is entirely an in vitro process, whereby selected leads are then progressed through a series of complex surrogate assays to evaluate efficacious bio-pharmacological traits, class/compound-specific toxicological properties and favourable absorption, distribution, metabolism, and excretion (ADME) properties [12]. It is in these early stages of testing that most compounds fail.

Further lead validation is through efficacy, ADME and toxicology testing in animal models. A drawback of this in vivo process is that the current short-term testing protocol employed primarily defines the toxicological profile rather than therapeutic efficacy. During this stage of the development process, scale-up methodologies for the use of the selected lead in clinical trials are tested. The clinical trial process itself can commence only after FDA approval of an Investigational New Drug (IND) application which details pre-clinical results, proposed mode of drug action, potential side effects and manufacturing information. The Phased clinical trials (I, II and III) are overseen by a clinical research team in close communication with the FDA, with Phase I focussing on drug safety, Phase II on effectivity, and Phase III on confirming the findings on a larger population cohort. A successful outcome in the clinical trials will lead to the submission of a New Drug Application for further scrutiny and approval by the FDA and other regulatory bodies.

## 3. Challenges to Drug Discovery

A key step in the innovative drug discovery process is the identification of a molecular lead which potently modulates the chosen target to produce a desirable pharmacological outcome which translates predictably to the human host [13]. The success of this process is reliant on two factors: Firstly, access to robust, scientific literature permitting the acquisition of available knowledge on the biological processes underlying health and disease; secondly, the availability of appropriate pre-clinical tests and model systems that permit the verification of safe, efficacious and translatable pharmacokinetic and pharmacodynamic (PK/PD) relationships. Although animal models have indeed proved predictive in many instances, inadequate testing for congruence with human disease has led to costly translational failures, and remains a significant deficit in the pre-clinical developmental phase of the drug discovery programme [3]. The extensive study by Seok et al., (2013) exemplifies this challenge in their report on the failure of mouse models in human inflammatory disease [14]. Models that can accurately mimic human disease and reliably characterize the longitudinal behaviour of clinical endpoints are urgently needed to minimise attrition and associated risk in the next phase of clinical development [15]. Appropriate biomarkers that represent these endpoints and those which enable better clinical phenotyping and patient stratification will be useful tools to validate pre-clinical assumptions more predictably [16]. Patient heterogeneity is a crucial factor contributing to failure at the clinical trial phase. Omics methodology could potentially offer new ways to stratify patients and enable the clustering of more homogeneous cohorts. This clearly must go beyond merely the simplistic identification of a gene polymorphism in association with a disease trait, and entail rather a more complex mapping of the wider association between genotype and associated phenotypes.

Recent times have witnessed a relentless focus on improving speed and efficacy of the drug development processes (e.g., first to market) in the hope of attaining fast financial returns. The reality, however, is a sharp decline in productivity with failure to recover returns on investment. Such initiatives, while important, have taken the focus off the quality of science, a critical starting point of the process. The driving impetus towards profitability and early returns has resulted in an over-reliance on high-throughput technical advances, before such technologies were allowed a maturation phase. The long-term returns and sustainability of the industry are more likely to be secured through initiatives focussing on the quality of the science underpinning the process, rather than on merely the speed and efficiency of the process [17]. Earlier (Pre-IND) engagement with regulatory bodies may significantly reduce the delays that occur at this stage of the process by enabling better communication and clarity on the process.

Justifiably, the industry has attempted to put in place wider measures to curb or cope with the spiralling declines in R&D productivity. The past decade has seen a significant increase in the outsourcing of discovery activities to low-cost locations like India and China where rapid expansions have occurred in the investment on academic and government-funded research sectors to maximise opportunities, particularly for diseases relevant to each country. Given the sharp contraction of late-stage pipelines, the extremely stringent pre-clinical screening criteria should indeed perhaps be reviewed. There is a significant likelihood that viable leads are disregarded very early in the process, purely on the basis of thresholds dictated by systems that are far from optimal in terms of fit for purpose.

## 4. Genomics in Drug Discovery

Traditionally, molecular targets for drug discovery were derived as a consequence of a lengthy “function to gene” process whereby a gene of interest derived from animal tissues was then cloned, expressed in a recombinant host and functionally characterised. The tedious and time-consuming process however, delivered specific targets whose functions were well characterised [18]. The successful completion of the human genome project in 2003 spurred on a revolution in biotechnological advances resulting in the diverse umbrella discipline of “Omics” as we know it today. The resultant high-throughput advances in sequencing led to the identification of thousands of genes, with little information on function and a paradigm shift to a “gene to screen” research approach [19]. Genomics outputs have already significantly contributed toward furthering our understanding of disease biology and diagnosis. The discovery of Cathepsin K as a molecular target for osteoporosis and the sequencing of all individual members of gene super-families (e.g., G protein-coupled receptors, ion channels, nuclear hormone receptors, proteases, kinases, etc.) have had significant implication for the drug discovery program [20,21]. The permeating influence of genomics on drug development underpins the pharmaceutical industry’s shift in focus from researching new chemistry to understanding underlying biology. Pharmacogenomics is an exciting new field that seeks to define genetic markers predicting individual responses to drugs. The sharp and progressive decline in the cost of sequencing genomes makes the technology readily accessible to researchers. The progressive reduction in the cost of sequencing and the consequent expansion of the sequencing project beyond the human genome has enabled comparative genomics to be exploited to identify pathogen-specific targets. The availability of sequence information for a large cohort (~50) of pathogenic and non-pathogenic bacterial genomes enables information on virulence mechanisms and the development of therapeutics specifically directed against the pathogen metabolic pathways [22]. The discovery of diarylquinolones targeting the mycobacterial ATP synthase, for example, demonstrated how sequence diversity can be exploited to target pathways perceived to be ubiquitous [23]. The science has also enabled a range of genetic manipulations (e.g., knockout studies, mutation analyses, the construction of conditional mutants, transcriptome profiling etc.) which will support drug discovery by not only increasing knowledge of gene function and pathogen virulence mechanisms, but also enable the development of genetically engineered animal models for drug discovery and toxicity testing.

From a future perspective, it is envisaged that the most significant impact from genomics applications will be seen in the clinical phase of the drug development pipeline. Improving clinical trial design by using objective genetic inclusion and exclusion criteria will enable improved stratification of disease and thereby the definition of a more homogeneous cohort of patients. Similarly, genetic markers for efficacy and toxicity could be important predictive signatures capable of pre-selecting trial cohorts inherently susceptible to non-target effects, thereby improving the costly late-phase failures in the pipeline.

## 5. Proteomics in Drug Discovery

The proteome refers to the entire protein complement expressed by a genome [24,25], and proteomics is the study of the molecular and cellular dynamics of global protein expression and function [26]. Proteomics presents formidable challenges for many reasons [27,28]. The proteome is a dynamic entity, subject to a high degree of post-translational modification with consequent functional implications. The science lacks the advantage of an amplification methodology akin to the polymerase chain reaction in genomics and, therefore, capturing the widely diverse protein concentration profiles in biological cellular and fluid environments continues to be challenging [29,30]. However, there is little doubt that this evolving technological platform holds considerable promise for deciphering the complex biological perturbations in disease states, enabling translation to effective, targeted, therapeutic interventions. The high-throughput technical advances that have ensued through the past decade permit the global and focussed capture of dynamic changes in complex proteomes in a quantitative manner. The coming of age of quantitative proteomic methodologies, together with increasing awareness of the pre-analytical and analytical variables that confound data interpretation, have ensured that modern proteomic applications are increasingly offering a viable route to delivering robust, reproducible and standardised data sets [31,32]. The differential and quantitative profiling of the dynamic protein changes in health and disease will inevitably further our understanding of the mechanistic basis of disease.

Pharmacoproteomics has been utilised in many areas of drug development, namely identification and validation of drug targets, informing assay development for screening of leads and in generating in vitro and in vivo biomarkers as surrogate endpoints for efficacy, toxicology and disease stratification [33,34]. The technology is adaptable to generating large-scale global data sets as well as more focussed, functional investigations related to specific pathways, and can be used to cross-corroborate data inferred from other Omics inputs including genomics, transcriptomics and metabolomics.

A typical proteomic approach involves protein extraction/fractionation or separation of proteins in a complex mixture, followed by identification through mass spectrometry and quantitation. Although considered the workhorse of proteomics, older separation methodologies like two-dimensional gel electrophoresis (2DGE) had several limitations from a drug discovery perspective. Its failure to detect proteins of low abundance, proteins at either extreme of size and isoelectric point and membrane proteins (representing 50% of drug targets), meant the exclusion of an important cohort of proteins involved in biological regulation [35,36] Much of the 2DGE separation methods have been overtaken by advances in the chromatography arena. Early micro-preparative high-performance liquid chromatography (HPLC) systems, though initially plagued by poor column recovery, were advanced very rapidly through to the diverse, modern, mass spectrometry-compatible Nano HPLC systems we have today, with much improved separation efficacies, excellent recoveries and improved reproducibility. Further advances in extraction and enrichment methodologies, where difficult fractions like the membrane (MS compatible detergent extraction e.g., Rapigest (Waters) and phospho-proteomes (e.g., titanium dioxide nanotrap columns) are better accessed, have successfully addressed issues arising from sample complexity, thus permitting deeper mining of subset proteomes at higher resolution and better reproducibility [28]. The lack of robust quantitative strategies in the early phase of proteomics, limited reliable standardisation, and comparison of methodologies further contributed to the poor reproducibility associated with the field. Here, too, the launch of newer generation, high-resolution, mass spectrometers like the quadrupole-orbitraps, by leveraging fast acquisition and high-resolving power and trapping capabilities, permitted novel parallel reaction monitoring (PRM) approaches for targeted quantitative proteomics. The new methodology, by enabling decoupling of data acquisition and data processing, offers unmatched levels of selectivity and sensitivity for the analysis of complex biological samples. While Data Independent Acquisition (DIA) strategies (e.g., SWATH-MS, MSE, etc.) have permitted the more widespread application of label-free strategies, a plethora of labelled quantitative methods (SILAC, ICAT, ITRAQ etc.) complement existing methods to achieve robust quantitative data set [37,38,39]. Furthermore, the imperative for rigorous standardisation and the need to systematically collate the multidisciplinary outputs in a more strategic, coordinated and integrated manner, led to the founding of the Human Proteome Organisation (HUPO) in 2001, with a mandate to identify and characterise at least one protein product from each of the 20,300 human protein encoding genes, including available data on associated post-translational modifications (PTMs), splice-variant isoforms and single-amino-acid polymorphisms. The maturation and standardisation of proteomics technology make it a versatile drug development tool to be incorporated into the discovery programme.

## 6. Metabolomics in Drug Discovery

Whilst genes code for proteins, it is the action of the proteins that ultimately affects the phenotype of the organism as the proteins engage in a range of complex metabolic reactions. Since the 1960s, chromatography methods have been utilised to separate and isolate these small molecules that are produced, degraded and transformed within the organism. The coupling of gas chromatography and mass spectrometry, alongside improvements in detectors, has allowed us to qualitatively and quantitatively assess all small molecular weight molecules (metabolites) within a cell, tissue or organism [40]. Techniques have been further refined in the last 15 years by the utilisation of more sophisticated separation techniques (capillary electrophoresis, liquid chromatography, etc.) and additional detection methods (nuclear magnetic resonance, Fourier transform infrared spectroscopy) [41]. Tens of thousands of metabolites have been identified in humans and are collected in the human metabolome database (www.hmdb.ca). Analytic techniques include widespread analysis of all detectable metabolites (so-called “fingerprinting”), or quantitative analysis of specific targets to answer a specific clinical question.

There are a number of roles that metabolomics may have in the development of NMEs into viable therapeutic medicines. One role may be in bridging the gap between animal and human studies. There is a large attrition rate when drugs move from animal models into human trials; this is in large part due to the differences between animal models and their human counterparts. Metabolomics has identified concordance with some murine models of diabetes and differences in others [42]. Identification of these differences is perhaps the first step in improving our animal models and making them more analogous to the human disease processes.

Another role for metabolomics is in clarifying disease processes. Novel small-molecular-weight compounds have been identified in association with atherosclerosis and diabetes, are produced by gut microflora, have never before been associated with the disease processes and represent novel targets for future therapeutic agents [43]. This approach has also been applied to cancer treatment. A number of metabolites have been identified as upregulated in certain cell lines. Experimental work to knock out the gene responsible for the enzyme producing the metabolite has had success in reducing tumour growth [44].

Metabolomics also has the potential for generating a new generation of biomarkers. The concept is that various disease states could be characterised by a specific metabolite or a pattern of metabolite changes and that these would be small molecules that are readily identifiable [43]. A number of models have been assessed including a number of cancers and inflammatory conditions. However, there has been limited success when trials have attempted to validate these novel biomarkers in clinical practice [45].

Drug safety may also be analysed by the application of a metabolomics approach. Considerable advances have been made in the assessment of mechanisms of action of toxicity of drugs and other substances. A database is currently being created that aims to map all known pathways of toxicity, specifically starting with endocrine disruptors (www.humantoxome.com). Unfortunately, such techniques are currently limited by a number of issues such as a lack of reproducibility and standardisation, meaning that an in vitro method of assessing toxicity with metabolomics profiles remains a goal for the future rather than a reality in the present [46].

## 7. Bioinformatics in Drug and Biomarker Discovery

The global analytical capabilities of Omics platforms result in large, complex “data-rich” outputs; their accurate processing is critical, tedious, costly and time consuming. The generation of ever-increasing data sets has necessitated concurrent development of bioinformatics methodology, not only to catalogue outputs but also to systematically analyse, compare and enable sophisticated computer-aided interpretations. Recent years have witnessed unprecedented increases in proprietary and free open-source software resources aimed at processing Omics-related data [43]. Storage and dissemination of processed data is greatly enabled by multiple databases including, Peptide Atlas [47], Human Protein Atlas [48,49], NeXtProt [50] etc., which aim to capture multi-source data with precise annotations, user-friendly web interfaces and a fully traceable data provenance. Recent trends towards implementation of open-access policies by leading journals and research councils will favour the open sharing and dissemination of such data. While there is still some way to go towards defining measurable metrics to evaluate the quality of the collated data and perhaps cross-validating across Omics specialities to enable a systems-biology approach, the progress thus far is impressive and encouraging.

## 8. Conclusions

Dissipating proprietary assets, diminishing pipelines and soaring R&D costs have forced a once-thriving pharmaceutical industry into “survival mode” [3,51]. Impending loss of proprietary protection on primary care blockbuster drugs from the 1990s’ “golden discovery era,” is likely to further dent projected revenues [51]. With the low-hanging fruit already picked, successful advancement of a lead compound to late-phase clinical development is increasingly challenging. The costly failures are largely attributed to loss of efficacy in the clinical phase of the development process, namely the Phase II and III trials [3]. Interestingly, the clinical attrition trends are changing too. The causative principle component for the clinical phase drug failures in the 1990s was attributed to unacceptable pharmacokinetic profiles in humans. In response to this, the industry introduced several refinements to pre-clinical testing modalities to improve issues, including allometric scaling for projection to human pharmacokinetics, in the areas of drug absorption, distribution, metabolism and excretion (ADME), with very successful outcomes. However, more recent clinical attrition data attributes failures to loss of efficacy and poor safety profiles [52,53]. This not only focusses the attention back on to early target selection/lead generation, but it also questions the suitability of current animal models with respect to congruency with and extrapolation of findings for human hosts. More recently, researchers have focussed on drug repositioning, whereby new uses are found for existing drugs, as a valid, alternative route to de-risk and improve the efficacy of the drug discovery process [8,54]. The approach which utilises bioactive compounds with defined safety profiles has already yielded interesting leads for a range of diseases including cancer, Alzheimer’s disease and malaria [8,54,55,56]. The availability of established clinical drug libraries (Library of Pharmacologically Active Compounds (LOPAC1280), the NIH Clinical Collection (NIHCC) etc.), with associated data on structure, function etc., permits not only assay and in silico-based screening, but also further structural modification to enhance pharmacological activity. The strategy of systematically scanning the existing pharmacopoeia permits early in vitro pharmacokinetic profiling [57] and, in libraries already approved by the Food and Drug Administration agency (FDA) or the European Medicines Agency (EMA), faster ADMET testing.

Return on R&D investment and the ensuing sustainability for the pharmaceutical industry is dependent on innovative processing and harnessing of information from powerful technological platforms already at its disposal. Interestingly, the vast majority of basic science inventions that translate into innovative medicines originate from academic laboratories [58,59,60]. However, industry and academia, the two major stakeholders that are essential game changers in this complicated conundrum have continued to work in isolation, driven by widely varying goals, overlooking the fact that their competencies and capabilities have little overlap and are in fact complementary. While industry continues to excel in processes pertaining to scale, high-throughput infrastructure and manufacture, academia is set to remain the source of new knowledge, derived through meticulous and time-consuming scientific enquiry. A partnership where risks and rewards are shared must surely be the way forward. The relentless pressure, both on industry and academia to achieve measurable outputs within limited time scales has failed to foster an environment that supports scientific creativity. Hence, opportunities for serendipitous discovery, which have in the past sustained the pharmaceutical industry are missed out [61,62,63,64]. If competitive differences can be overlooked in favour of collaborative initiatives, particularly in the discovery phase of the drug development process, the crucial target selection and lead generation element of the discovery will be enabled, resulting in a de-risked approach with higher successful completion rates in the discovery pipeline.

We are now entering an era where progressively maturating Omics-based technologies are poised to deliver on long-promised targets pertaining to the improved understanding of the complex mechanistic basis of disease. Arguably, the paradigm shift from a traditional “hypothesis-driven” research environment to one that is primarily “discovery-based” will fail to sit comfortably with many researchers. The integrative analysis of the large data sets churned out continues to prove a challenge, with advances in analytical methodology failing to keep abreast of technical advances. Nevertheless, for the first time ever, an integrated approach to modelling and defining the immense complexities of health and disease is emerging. Its implications are likely to transcend far beyond improving our mechanistic understanding of health and disease or drug and biomarker discovery. We are already seeing the heralding of the arrival of personalised medicine and a paradigm shift in the focus from disease to health. Clearly, the ultimate realisation of such revolutionary visions and concepts will predictably entail many obstacles and hurdles from experimental, technical, analytical and financial viewpoints. The key to future drug discovery will reside in our ability to harness the powerful new technologies already at our disposal to integrate information from sequenced genomes, functional genomics, protein profiling, metabolomics and bioinformatics, in a manner that ensures a comprehensive systems-based analysis to further our understanding of the complexities of health and disease.
